# Supplementary material for: Phylogeographic structure in three North American tent caterpillar species (Lepidoptera: Lasiocampidae): Malacosoma americana, M. californica, and M. disstria
Source: PeerJ. 2018 Mar 19;6:e4479. doi: 10.7717/peerj.4479 (PMC5863710; doi:10.7717/peerj.4479)
Supplement: Table S1 — Sample information for five North American Malacosoma species. The BOLD sample ID, GenBank Accession Number, and sampling location are given. GPS coordinates for M. americana, M. californica, and M. disstria are given where available. [file peerj-06-4479-s001.docx]

| **Species Name** | **BOLD ID** | **GenBank ID** | **Country** | **Province/State** | **Lat** | **Long** |
| --- | --- | --- | --- | --- | --- | --- |
| ***North America*** |  |  |  |  |  |  |
| *M. americana* | HBL008654 | GU095228 | Canada | New Brunswick | 45.08 | -67.07 |
| *M. americana* | HBL008656 | GU095229 | Canada | New Brunswick | 45.08 | -67.07 |
| *M. americana* | moth85.02SA | GU438462 | Canada | New Brunswick | 45.08 | -67.07 |
| *M. americana* | 04HBL007662 | GU438459 | Canada | New Brunswick | 45.08 | -67.07 |
| *M. americana* | 04HBL007665 | GU438460 | Canada | New Brunswick | 45.08 | -67.07 |
| *M. americana* | 04HBL007672 | GU438461 | Canada | New Brunswick | 45.08 | -67.07 |
| *M. americana* | MNBTT-038 | KT143906 | Canada | New Brunswick | 46.00 | -66.18 |
| *M. americana* | MNBTT-239 | KT145755 | Canada | New Brunswick | 45.85 | -67.70 |
| *M. americana* | MNBTT-910 | KT127209 | Canada | New Brunswick | 46.00 | -66.18 |
| *M. americana* | MNBTT-912 | KT145097 | Canada | New Brunswick | 46.00 | -66.18 |
| *M. americana* | MNBTT-913 | KT134477 | Canada | New Brunswick | 46.00 | -66.18 |
| *M. americana* | CNCLEP 92354 | n/a | Canada | New Brunswick | n/a | n/a |
| *M. americana* | moth943.02 | GU094642 | Canada | Ontario | 43.54 | -80.13 |
| *M. americana* | moth953.02 | GU094643 | Canada | Ontario | 43.54 | -80.13 |
| *M. americana* | 04HBL005085 | GU094063 | Canada | Ontario | 43.54 | -80.13 |
| *M. americana* | 04HBL005092 | GU094062 | Canada | Ontario | 43.54 | -80.13 |
| *M. americana* | 04HBL005098 | GU094064 | Canada | Ontario | 43.54 | -80.13 |
| *M. americana* | 04HBL005148 | GU094065 | Canada | Ontario | 43.54 | -80.13 |
| *M. americana* | 04HBL005180 | GU094066 | Canada | Ontario | 43.54 | -80.13 |
| *M. americana* | 2005-ONT-553 | GU438991 | Canada | Ontario | 44.53 | -77.00 |
| *M. americana* | 2005-ONT-559 | GU438992 | Canada | Ontario | 44.53 | -77.00 |
| *M. americana* | 2005-ONT-566 | GU438993 | Canada | Ontario | 44.53 | -77.00 |
| *M. americana* | 2005-ONT-580 | GU438995 | Canada | Ontario | 44.53 | -77.00 |
| *M. americana* | 2005-ONT-581 | GU438994 | Canada | Ontario | 44.53 | -77.00 |
| *M. americana* | 2005-ONT-582 | GU438996 | Canada | Ontario | 44.53 | -77.00 |
| *M. americana* | Moth4373.03 | GU092456 | Canada | Ontario | 43.54 | -80.13 |
| *M. americana* | Moth4384.03 | GU092458 | Canada | Ontario | 43.54 | -80.13 |
| *M. americana* | Moth4385.03 | GU092457 | Canada | Ontario | 43.54 | -80.13 |
| *M. americana* | Moth4386.03 | GU092455 | Canada | Ontario | 43.54 | -80.13 |
| *M. americana* | Moth4387.03 | GU092459 | Canada | Ontario | 43.54 | -80.13 |
| *M. americana* | Moth4388.03 | GU092460 | Canada | Ontario | 43.54 | -80.13 |
| *M. americana* | Moth4517.03 | GU092461 | Canada | Ontario | 43.54 | -80.13 |
| *M. americana* | Moth4537.03 | GU092463 | Canada | Ontario | 43.54 | -80.13 |
| *M. americana* | Moth4538.03 | GU092462 | Canada | Ontario | 43.54 | -80.13 |
| *M. americana* | Moth4578.03 | GU092464 | Canada | Ontario | 43.54 | -80.13 |
| *M. americana* | Moth4601.03 | GU092466 | Canada | Ontario | 43.54 | -80.13 |
| *M. americana* | Moth4602.03 | GU092465 | Canada | Ontario | 43.54 | -80.13 |
| *M. americana* | Moth4607.03 | GU092467 | Canada | Ontario | 43.54 | -80.13 |
| *M. americana* | 2006-ONT-0845 | KT142112 | Canada | Ontario | 44.53 | -77.00 |
| *M. americana* | 2006-ONT-0851 | KT144358 | Canada | Ontario | 44.53 | -77.00 |
| *M. americana* | 2006-ONT-0921 | KT139818 | Canada | Ontario | 44.53 | -77.00 |
| *M. americana* | 2006-ONT-1439 | KT134519 | Canada | Ontario | 44.53 | -77.00 |
| *M. americana* | 2006-ONT-1440 | KT134924 | Canada | Ontario | 44.53 | -77.00 |
| *M. americana* | BIOUG10886-C01 | n/a | Canada | Ontario | n/a | n/a |
| *M. americana* | BL481 | n/a | Canada | Ontario | n/a | n/a |
| *M. americana* | BIOUG10886-B12 | n/a | Canada | Ontario | n/a | n/a |
| *M. americana* | BIOUG10886-B11 | n/a | Canada | Ontario | n/a | n/a |
| *M. americana* | BIOUG10886-C02 | n/a | Canada | Ontario | n/a | n/a |
| *M. americana* | BL568 | n/a | Canada | Ontario | n/a | n/a |
| *M. americana* | BL1205 | n/a | Canada | Ontario | n/a | n/a |
| *M. americana* | BIOUG10886-C03 | n/a | Canada | Ontario | n/a | n/a |
| *M. americana* | DH009351 | n/a | Canada | Quebec | 45.65 | -72.45 |
| *M. americana* | 09BBLEP-04423 | GU694717 | United States | Arkansas | 34.84 | -91.47 |
| *M. americana* | 10BBLEP-00511 | HQ985858 | United States | Arkansas | 35.37 | -93.34 |
| *M. americana* | SNS10IL-00235 | n/a | United States | Illinois | 38.23 | -88.19 |
| *M. americana* | 06-SUSA-0190 | n/a | United States | Kentucky | 37.01 | -88.54 |
| *M. americana* | AM-94-0145 | n/a | United States | Maryland | 39.13 | -76.88 |
| *M. americana* | BIOUG01047-E02 | n/a | United States | Minnesota | 47.00 | -94.69 |
| *M. americana* | BIOUG01047-E08 | n/a | United States | Minnesota | 47.00 | -94.69 |
| *M. americana* | BIOUG01047-H08 | n/a | United States | Minnesota | 47.00 | -94.69 |
| *M. americana* | 05-NCCC-217 | n/a | United States | North Carolina | 34.88 | -76.94 |
| *M. americana* | 06-NCC-1038 | n/a | United States | North Carolina | 34.77 | -76.76 |
| *M. americana* | 09BBLEP-01466 | HM426913 | United States | Oklahoma | 33.88 | -96.81 |
| *M. americana* | 09BBLEP-01598 | HM427040 | United States | Oklahoma | 33.88 | -96.81 |
| *M. americana* | 09BBLEP-01735 | HM427173 | United States | Oklahoma | 33.88 | -96.84 |
| *M. americana* | 09BBLEP-02036 | HM427470 | United States | Oklahoma | 33.88 | -96.82 |
| *M. americana* | 09BBLEP-02037 | HM427471 | United States | Oklahoma | 33.88 | -96.82 |
| *M. americana* | 09BBLEP-02102 | HM427536 | United States | Oklahoma | 33.88 | -96.82 |
| *M. americana* | 09BBLEP-03013 | GU690763 | United States | Oklahoma | 33.88 | -96.80 |
| *M. americana* | 09BBLEP-01734 | HM427172 | United States | Oklahoma | 33.88 | -96.84 |
| *M. americana* | MDOK-1378 | n/a | United States | Oklahoma | 36.74 | -95.95 |
| *M. americana* | DNA-ATBI-2699 | GU088989 | United States | Tennessee | 35.71 | -83.65 |
| *M. americana* | DNA-ATBI-2700 | GU088990 | United States | Tennessee | 35.69 | -83.65 |
| *M. americana* | DNA-ATBI-2701 | GU088991 | United States | Tennessee | 35.75 | -83.21 |
| *M. americana* | 05-TN-00324 | GU087416 | United States | Tennessee | 35.46 | -83.21 |
| *M. americana* | TAMUICEGR-0823 | HQ978003 | United States | Texas | 30.59 | -96.25 |
| *M. americana* | TAMUICEGR-0824 | HQ978004 | United States | Texas | 30.59 | -96.25 |
| *M. americana* | TAMUICEGR-0825 | HQ978005 | United States | Texas | 35.54 | -101.73 |
| *M. americana* | TAMUICEGR-0826 | HQ978006 | United States | Texas | 35.54 | -101.73 |
| *M. californica* | BIOUG03610-E07 | KM543079 | Canada | Alberta |  |  |
| *M. californica* | BIOUG07103-B07 | KM540666 | Canada | Alberta |  |  |
| *M. californica* | BIOUG07103-C09 | KM542808 | Canada | Alberta |  |  |
| *M. californica* | 08BBLEP-02581 | KM549219 | Canada | Alberta |  |  |
| *M. californica* | 08BBLEP-02574 | KM552005 | Canada | Alberta |  |  |
| *M. californica* | 08BBLEP-02580 | KM554205 | Canada | Alberta |  |  |
| *M. californica* | 08BBLEP-04944 | KM540650 | Canada | Alberta |  |  |
| *M. californica* | 08BBLEP-02255 | KM550577 | Canada | Alberta |  |  |
| *M. californica* | 08BBLEP-02269 | KM540710 | Canada | Alberta |  |  |
| *M. californica* | CNCNoctuoidea13837 | n/a | Canada | Alberta |  |  |
| *M. californica* | CNCNoctuoidea13838 | n/a | Canada | Alberta |  |  |
| *M. californica* | 10BBCLP-0422 | JF841458 | Canada | British Columbia | 50.63 | -116.06 |
| *M. californica* | 10BBCLP-0423 | JF841459 | Canada | British Columbia | 50.99 | -118.16 |
| *M. californica* | 10BBCLP-0425 | JF841461 | Canada | British Columbia | 50.99 | -118.16 |
| *M. californica* | 10BBCLP-0426 | JF841462 | Canada | British Columbia | 50.99 | -118.16 |
| *M. californica* | 10BBCLP-0427 | JF841463 | Canada | British Columbia | 50.99 | -118.16 |
| *M. californica* | 10BBCLP-0431 | JF841467 | Canada | British Columbia | 50.99 | -118.16 |
| *M. californica* | 10BBCLP-0432 | JF841468 | Canada | British Columbia | 50.99 | -118.16 |
| *M. californica* | 10BBCLP-0433 | JF841469 | Canada | British Columbia | 50.99 | -118.16 |
| *M. californica* | 10BBCLP-0421 | JF841457 | Canada | British Columbia | 50.63 | -116.06 |
| *M. californica* | 10BBCLP-0428 | JF841464 | Canada | British Columbia | 50.99 | -118.16 |
| *M. californica* | BIOUG03567-G03 | KM553605 | Canada | British Columbia | 48.67 | -123.43 |
| *M. californica* | BIOUG03567-G04 | KM540642 | Canada | British Columbia | 48.67 | -123.43 |
| *M. californica* | BIOUG03567-G05 | KM546389 | Canada | British Columbia | 48.67 | -123.43 |
| *M. californica* | BIOUG03567-G06 | KM550451 | Canada | British Columbia | 48.67 | -123.43 |
| *M. californica* | BIOUG03567-G07 | KM552895 | Canada | British Columbia | 48.67 | -123.43 |
| *M. californica* | BIOUG03567-G08 | KM552555 | Canada | British Columbia | 48.67 | -123.43 |
| *M. californica* | BIOUG03567-G09 | KM547343 | Canada | British Columbia | 48.67 | -123.43 |
| *M. californica* | BIOUG03567-G10 | KM540759 | Canada | British Columbia | 48.67 | -123.43 |
| *M. californica* | BIOUG03608-A06 | KM539575 | Canada | British Columbia | 48.67 | -123.43 |
| *M. californica* | BIOUG07571-B03 | KM541216 | Canada | British Columbia | 48.67 | -123.43 |
| *M. californica* | BIOUG07571-B04 | KM548101 | Canada | British Columbia | 48.67 | -123.43 |
| *M. californica* | BIOUG04567-G10 | KM548398 | Canada | British Columbia | 51.02 | -118.21 |
| *M. californica* | BIOUG18909-H11 | KR936651 | Canada | British Columbia | 51.37 | -116.53 |
| *M. californica* | AVBC 332-10 | JF852765 | Canada | British Columbia | 49.30 | -124.78 |
| *M. californica* | AVBC 365-10 | JF852798 | Canada | British Columbia | 49.30 | -124.78 |
| *M. californica* | AVBC 1158-11 | KT134638 | Canada | British Columbia | 50.08 | -122.98 |
| *M. californica* | HLC-20813 | KT144177 | Canada | British Columbia | 51.02 | -118.21 |
| *M. californica* | HLC-20814 | KT128884 | Canada | British Columbia | 51.02 | -118.21 |
| *M. californica* | HLC-20815 | KT141100 | Canada | British Columbia | 51.02 | -118.21 |
| *M. californica* | HLC-20816 | KT134202 | Canada | British Columbia | 51.02 | -118.21 |
| *M. californica* | HLC-20817 | KT145243 | Canada | British Columbia | 51.02 | -118.21 |
| *M. californica* | HLC-20818 | KT136083 | Canada | British Columbia | 51.02 | -118.21 |
| *M. californica* | HLC-20819 | KT132405 | Canada | British Columbia | 51.02 | -118.21 |
| *M. californica* | HLC-20820 | KT141539 | Canada | British Columbia | 51.02 | -118.21 |
| *M. californica* | HLC-20821 | KT125026 | Canada | British Columbia | 51.02 | -118.21 |
| *M. californica* | HLC-20822 | KT126344 | Canada | British Columbia | 51.02 | -118.21 |
| *M. californica* | HLC-20823 | KT132116 | Canada | British Columbia | 51.02 | -118.21 |
| *M. californica* | HLC-20824 | KT143658 | Canada | British Columbia | 51.02 | -118.21 |
| *M. californica* | HLC-21018 | KT147195 | Canada | British Columbia | 51.01 | -118.19 |
| *M. californica* | HLC-21128 | KT137160 | Canada | British Columbia | 51.02 | -118.21 |
| *M. californica* | HLC-21129 | KT146833 | Canada | British Columbia | 51.02 | -118.21 |
| *M. californica* | HLC-21130 | KT135407 | Canada | British Columbia | 51.02 | -118.21 |
| *M. californica* | HLC-21132 | KT134747 | Canada | British Columbia | 51.02 | -118.21 |
| *M. californica* | HLC-21133 | KT125882 | Canada | British Columbia | 51.02 | -118.21 |
| *M. californica* | HLC-21136 | KT144366 | Canada | British Columbia | 51.02 | -118.21 |
| *M. californica* | HLC-21431 | KT136587 | Canada | British Columbia | 51.30 | -117.52 |
| *M. californica* | HLC-21520 | KT126253 | Canada | British Columbia | 51.30 | -117.52 |
| *M. californica* | HLC-21568 | KT128908 | Canada | British Columbia | 51.02 | -118.21 |
| *M. californica* | HLC-21862 | KT126095 | Canada | British Columbia | 51.09 | -117.92 |
| *M. californica* | HLC-21863 | KT135858 | Canada | British Columbia | 51.09 | -117.92 |
| *M. californica* | HLC-21864 | KT127336 | Canada | British Columbia | 51.09 | -117.92 |
| *M. californica* | HLC-21865 | KT134578 | Canada | British Columbia | 51.09 | -117.92 |
| *M. californica* | HLC-21866 | KT139358 | Canada | British Columbia | 51.09 | -117.92 |
| *M. californica* | HLC-21867 | KT125203 | Canada | British Columbia | 51.09 | -117.92 |
| *M. californica* | HLC-21868 | KT143080 | Canada | British Columbia | 51.09 | -117.92 |
| *M. californica* | HLC-21869 | KT144824 | Canada | British Columbia | 51.09 | -117.92 |
| *M. californica* | HLC-21870 | KT141651 | Canada | British Columbia | 51.09 | -117.92 |
| *M. californica* | HLC-21871 | KT135651 | Canada | British Columbia | 51.09 | -117.92 |
| *M. californica* | HLC-21872 | KT142698 | Canada | British Columbia | 51.09 | -117.92 |
| *M. californica* | HLC-21873 | KT144155 | Canada | British Columbia | 51.09 | -117.92 |
| *M. californica* | HLC-21874 | KT145445 | Canada | British Columbia | 51.09 | -117.92 |
| *M. californica* | HLC-21876 | KT132182 | Canada | British Columbia | 51.09 | -117.92 |
| *M. californica* | HLC-22222 | KT136439 | Canada | British Columbia | 51.01 | -118.19 |
| *M. californica* | HLC-22324 | KT139104 | Canada | British Columbia | 51.02 | -118.21 |
| *M. californica* | HLC-22325 | KT135394 | Canada | British Columbia | 51.02 | -118.21 |
| *M. californica* | HLC-22326 | KT141471 | Canada | British Columbia | 51.02 | -118.21 |
| *M. californica* | HLC-22327 | KT135776 | Canada | British Columbia | 51.02 | -118.21 |
| *M. californica* | HLC-22328 | KT142889 | Canada | British Columbia | 51.02 | -118.21 |
| *M. californica* | HLC-22329 | KT146444 | Canada | British Columbia | 51.02 | -118.21 |
| *M. californica* | HLC-22330 | KT140724 | Canada | British Columbia | 51.02 | -118.21 |
| *M. californica* | HLC-22423 | KT145647 | Canada | British Columbia | 51.30 | -117.52 |
| *M. californica* | HLC-22424 | KT125332 | Canada | British Columbia | 51.30 | -117.52 |
| *M. californica* | HLC-22425 | KT137982 | Canada | British Columbia | 51.30 | -117.52 |
| *M. californica* | HLC-22426 | KT134902 | Canada | British Columbia | 51.30 | -117.52 |
| *M. californica* | HLC-22428 | KT133465 | Canada | British Columbia | 51.30 | -117.52 |
| *M. californica* | HLC-22429 | KT142839 | Canada | British Columbia | 51.30 | -117.52 |
| *M. californica* | HLC-22430 | KT147905 | Canada | British Columbia | 51.30 | -117.52 |
| *M. californica* | HLC-22431 | KT142525 | Canada | British Columbia | 51.30 | -117.52 |
| *M. californica* | HLC-22432 | KT136463 | Canada | British Columbia | 51.30 | -117.52 |
| *M. californica* | HLC-22433 | KT139266 | Canada | British Columbia | 51.30 | -117.52 |
| *M. californica* | HLC-22434 | KT134962 | Canada | British Columbia | 51.30 | -117.52 |
| *M. californica* | HLC-22510 | KT130264 | Canada | British Columbia | 51.36 | -117.43 |
| *M. californica* | HLC-22511 | KT132448 | Canada | British Columbia | 51.36 | -117.43 |
| *M. californica* | HLC-22513 | KT126148 | Canada | British Columbia | 51.36 | -117.43 |
| *M. californica* | HLC-22515 | KT128710 | Canada | British Columbia | 51.36 | -117.43 |
| *M. californica* | HLC-22516 | KT131235 | Canada | British Columbia | 51.36 | -117.43 |
| *M. californica* | HLC-22517 | KT145718 | Canada | British Columbia | 51.36 | -117.43 |
| *M. californica* | HLC-22544 | KT143549 | Canada | British Columbia | 51.30 | -117.52 |
| *M. californica* | HLC-22545 | KT128521 | Canada | British Columbia | 51.30 | -117.52 |
| *M. californica* | HLC-22546 | KT140709 | Canada | British Columbia | 51.30 | -117.52 |
| *M. californica* | HLC-22547 | KT132488 | Canada | British Columbia | 51.30 | -117.52 |
| *M. californica* | HLC-22548 | KT131854 | Canada | British Columbia | 51.30 | -117.52 |
| *M. californica* | HLC-22736 | KT139403 | Canada | British Columbia | 51.30 | -117.52 |
| *M. californica* | HLC-22737 | KT143086 | Canada | British Columbia | 51.30 | -117.52 |
| *M. californica* | HLC-22738 | KT132348 | Canada | British Columbia | 51.30 | -117.52 |
| *M. californica* | HLC-22739 | KT130320 | Canada | British Columbia | 51.30 | -117.52 |
| *M. californica* | HLC-22740 | KT146433 | Canada | British Columbia | 51.30 | -117.52 |
| *M. californica* | HLC-22741 | KT136198 | Canada | British Columbia | 51.30 | -117.52 |
| *M. californica* | HLC-22742 | KT139853 | Canada | British Columbia | 51.30 | -117.52 |
| *M. californica* | HLC-22743 | KT130581 | Canada | British Columbia | 51.30 | -117.52 |
| *M. californica* | HLC-22744 | KT143420 | Canada | British Columbia | 51.30 | -117.52 |
| *M. californica* | HLC-22745 | KT132398 | Canada | British Columbia | 51.30 | -117.52 |
| *M. californica* | HLC-22746 | KT138226 | Canada | British Columbia | 51.30 | -117.52 |
| *M. californica* | HLC-22747 | KT138610 | Canada | British Columbia | 51.30 | -117.52 |
| *M. californica* | HLC-22800 | KT147565 | Canada | British Columbia | 51.09 | -117.92 |
| *M. californica* | HLC-22801 | KT137904 | Canada | British Columbia | 51.09 | -117.92 |
| *M. californica* | HLC-22802 | KT131930 | Canada | British Columbia | 51.09 | -117.92 |
| *M. californica* | HLC-22803 | KT136891 | Canada | British Columbia | 51.09 | -117.92 |
| *M. californica* | HLC-22804 | KT135323 | Canada | British Columbia | 51.09 | -117.92 |
| *M. californica* | HLC-22805 | KT145468 | Canada | British Columbia | 51.09 | -117.92 |
| *M. californica* | HLC-22806 | KT127625 | Canada | British Columbia | 51.09 | -117.92 |
| *M. californica* | HLC-22807 | KT126333 | Canada | British Columbia | 51.09 | -117.92 |
| *M. californica* | HLC-22808 | KT125777 | Canada | British Columbia | 51.09 | -117.92 |
| *M. californica* | HLC-22809 | KT128935 | Canada | British Columbia | 51.09 | -117.92 |
| *M. californica* | HLC-22810 | KT127868 | Canada | British Columbia | 51.09 | -117.92 |
| *M. californica* | HLC-22811 | KT148429 | Canada | British Columbia | 51.09 | -117.92 |
| *M. californica* | HLC-22812 | KT131485 | Canada | British Columbia | 51.09 | -117.92 |
| *M. californica* | HLC-22813 | KT137531 | Canada | British Columbia | 51.09 | -117.92 |
| *M. californica* | HLC-22814 | KT133214 | Canada | British Columbia | 51.09 | -117.92 |
| *M. californica* | HLC-22815 | KT146685 | Canada | British Columbia | 51.09 | -117.92 |
| *M. californica* | HLC-22816 | KT134124 | Canada | British Columbia | 51.09 | -117.92 |
| *M. californica* | HLC-22817 | KT129549 | Canada | British Columbia | 51.09 | -117.92 |
| *M. californica* | HLC-22818 | KT144521 | Canada | British Columbia | 51.09 | -117.92 |
| *M. californica* | HLC-22820 | KT145773 | Canada | British Columbia | 51.09 | -117.92 |
| *M. californica* | HLC-22821 | KT125468 | Canada | British Columbia | 51.09 | -117.92 |
| *M. californica* | HLC-22822 | KT138845 | Canada | British Columbia | 51.09 | -117.92 |
| *M. californica* | HLC-22823 | KT139541 | Canada | British Columbia | 51.09 | -117.92 |
| *M. californica* | HLC-22824 | KT135999 | Canada | British Columbia | 51.09 | -117.92 |
| *M. californica* | HLC-22825 | KT126825 | Canada | British Columbia | 51.09 | -117.92 |
| *M. californica* | HLC-22826 | KT140472 | Canada | British Columbia | 51.09 | -117.92 |
| *M. californica* | HLC-22827 | KT126640 | Canada | British Columbia | 51.09 | -117.92 |
| *M. californica* | HLC-22828 | KT144958 | Canada | British Columbia | 51.09 | -117.92 |
| *M. californica* | HLC-22829 | KT127258 | Canada | British Columbia | 51.09 | -117.92 |
| *M. californica* | HLC-22830 | KT145065 | Canada | British Columbia | 51.09 | -117.92 |
| *M. californica* | HLC-22831 | KT129737 | Canada | British Columbia | 51.09 | -117.92 |
| *M. californica* | HLC-22832 | KT147968 | Canada | British Columbia | 51.09 | -117.92 |
| *M. californica* | HLC-22833 | KT125531 | Canada | British Columbia | 51.09 | -117.92 |
| *M. californica* | HLC-22927 | KT141891 | Canada | British Columbia | 51.26 | -117.55 |
| *M. californica* | HLC-22971 | KT128489 | Canada | British Columbia | 51.30 | -117.52 |
| *M. californica* | HLC-22972 | KT131229 | Canada | British Columbia | 51.30 | -117.52 |
| *M. californica* | HLC-22973 | KT143416 | Canada | British Columbia | 51.30 | -117.52 |
| *M. californica* | HLC-22974 | KT145563 | Canada | British Columbia | 51.30 | -117.52 |
| *M. californica* | HLC-22975 | KT133365 | Canada | British Columbia | 51.30 | -117.52 |
| *M. californica* | HLC-23029 | KT145930 | Canada | British Columbia | 51.30 | -117.52 |
| *M. californica* | HLC-23030 | KT137466 | Canada | British Columbia | 51.30 | -117.52 |
| *M. californica* | HLC-23031 | KT133795 | Canada | British Columbia | 51.30 | -117.52 |
| *M. californica* | HLC-23032 | KT133636 | Canada | British Columbia | 51.30 | -117.52 |
| *M. californica* | HLC-23033 | KT129608 | Canada | British Columbia | 51.30 | -117.52 |
| *M. californica* | HLC-23034 | KT132246 | Canada | British Columbia | 51.30 | -117.52 |
| *M. californica* | HLC-23288 | KT146816 | Canada | British Columbia | 51.35 | -117.44 |
| *M. californica* | HLC-23289 | KT141206 | Canada | British Columbia | 51.35 | -117.44 |
| *M. californica* | HLC-23290 | KT136527 | Canada | British Columbia | 51.35 | -117.44 |
| *M. californica* | HLC-23291 | KT145861 | Canada | British Columbia | 51.35 | -117.44 |
| *M. californica* | HLC-23293 | KT126249 | Canada | British Columbia | 51.35 | -117.44 |
| *M. californica* | 08-JDWBC-0640 | HQ683304 | Canada | British Columbia | 50.82 | -118.87 |
| *M. californica* | 10-JDWBC-2205 | HM863952 | Canada | British Columbia | 50.82 | -118.86 |
| *M. californica* | 10-JDWBC-6216 | HM868320 | Canada | British Columbia | 49.31 | -119.50 |
| *M. californica* | 10-JDWBC-6535 | HM868655 | Canada | British Columbia | 50.64 | -120.45 |
| *M. californica* | CGWC-0493 | KT141172 | Canada | British Columbia | 51.92 | -122.30 |
| *M. californica* | CGWC-0494 | KT141536 | Canada | British Columbia | 51.91 | -122.53 |
| *M. californica* | CGWC-0496 | KT130262 | Canada | British Columbia | 51.97 | -122.40 |
| *M. californica* | CGWC-0497 | KT130998 | Canada | British Columbia | 51.99 | -122.45 |
| *M. californica* | CGWC-0498 | KT131085 | Canada | British Columbia | 52.58 | -122.20 |
| *M. californica* | CGWC-0499 | KT128900 | Canada | British Columbia | 51.97 | -122.40 |
| *M. californica* | CGWC-0500 | KT138018 | Canada | British Columbia | 52.58 | -122.20 |
| *M. californica* | 08-JDWBC-1300 | n/a | Canada | British Columbia | 50.64 | -120.45 |
| *M. californica* | 08-JDWBC-1068 | n/a | Canada | British Columbia | 49.32 | -119.51 |
| *M. californica* | UBC-2006-1125 | n/a | Canada | British Columbia | 49.27 | -122.64 |
| *M. californica* | UBC-2006-1126 | n/a | Canada | British Columbia | 49.27 | -122.64 |
| *M. californica* | UBC-2006-1167 | n/a | Canada | British Columbia | 49.27 | -122.64 |
| *M. californica* | PFC-2006-0819 | n/a | Canada | British Columbia | 48.52 | -123.44 |
| *M. californica* | PFC-2006-0820 | n/a | Canada | British Columbia | 48.52 | -123.44 |
| *M. californica* | PFC-2006-0785 | n/a | Canada | British Columbia | 48.52 | -123.44 |
| *M. californica* | PFC-2006-2393 | n/a | Canada | British Columbia | 48.46 | -123.40 |
| *M. californica* | PFC-2006-2399 | n/a | Canada | British Columbia | 48.46 | -123.40 |
| *M. californica* | PFC-2006-2509 | n/a | Canada | British Columbia | 48.46 | -123.40 |
| *M. californica* | PFC-2006-2510 | n/a | Canada | British Columbia | 48.46 | -123.40 |
| *M. californica* | PFC-2006-2512 | n/a | Canada | British Columbia | 48.46 | -123.40 |
| *M. californica* | PFC-2006-2531 | n/a | Canada | British Columbia | 48.46 | -123.40 |
| *M. californica* | PFC-2006-2532 | n/a | Canada | British Columbia | 48.46 | -123.40 |
| *M. californica* | PFC-2006-2536 | n/a | Canada | British Columbia | 48.46 | -123.40 |
| *M. californica* | PFC-2006-2557 | n/a | Canada | British Columbia | 48.46 | -123.40 |
| *M. californica* | CNCNoctuoidea13832 | n/a | Canada | British Columbia | n/a | n/a |
| *M. californica* | Moth 410.03SA | GU438463 | Canada | New Brunswick |  |  |
| *M. californica* | BIOUG20637-D06 | n/a | Canada | Saskatchewan |  |  |
| *M. californica* | BIOUG20637-D08 | n/a | Canada | Saskatchewan |  |  |
| *M. californica* | BIOUG21136-D07 | n/a | Canada | Saskatchewan |  |  |
| *M. californica* | BIOUG21136-D08 | n/a | Canada | Saskatchewan |  |  |
| *M. californica* | CCDB-22945-E12 | n/a | United States | Arizona | n/a | n/a |
| *M. californica* | CCDB-22945-F01 | n/a | United States | Arizona | n/a | n/a |
| *M. californica* | BIOUG10886-C04 | n/a | United States | Arizona | n/a | n/a |
| *M. californica* | BIOUG10886-C05 | n/a | United States | Arizona | n/a | n/a |
| *M. californica* | CCDB-19575-C12 | n/a | United States | Arizona | n/a | n/a |
| *M. californica* | CNCLEP00067965 | GU679296 | United States | Arizona | 31.71 | -110.88 |
| *M. californica* | CNCLEP 81951 | n/a | United States | Arizona | 32.44 | -110.79 |
| *M. californica* | CNCLEP 81952 | n/a | United States | Arizona | 32.44 | -110.79 |
| *M. californica* | CNCLEP 81953 | n/a | United States | Arizona | 32.44 | -110.79 |
| *M. californica* | CNCLEP 81955 | n/a | United States | Arizona | 32.42 | -110.74 |
| *M. californica* | CCDB-22945-E05 | n/a | United States | California | n/a | n/a |
| *M. californica* | CCDB-22945-E06 | n/a | United States | California | n/a | n/a |
| *M. californica* | CCDB-22945-E07 | n/a | United States | California | n/a | n/a |
| *M. californica* | CNCLEP 81958 | n/a | United States | California | 34.34 | -116.84 |
| *M. californica* | BIOUG00848-G03 | JN279715 | United States | California | 40.32 | -120.99 |
| *M. californica* | BIOUG01541-A03 | n/a | United States | California | 37.97 | -119.12 |
| *M. californica* | BIOUG01541-A10 | n/a | United States | California | 37.97 | -119.12 |
| *M. californica* | BIOUG01541-B01 | n/a | United States | California | 37.97 | -119.12 |
| *M. californica* | CCDB-23268-B09 | n/a | United States | California | n/a | n/a |
| *M. californica* | CCDB-23268-B11 | n/a | United States | California | n/a | n/a |
| *M. californica* | BIOUG01453-D10 | n/a | United States | Texas | 34.97 | -101.67 |
| *M. californica* | RWWA-3664 | n/a | United States | Washington | 46.62 | -123.95 |
| *M. californica* | RWWA-2675 | n/a | United States | Washington | 46.62 | -123.95 |
| *M. californica* | RWWA-2693 | n/a | United States | Washington | 46.62 | -123.95 |
| *M. californica pluviale* | HLC-22512 | KT126669 | Canada | British Columbia |  |  |
| *M. californica pluviale* | HLC-22514 | KT146075 | Canada | British Columbia |  |  |
| *M. californica pluviale* | HLC-22819 | KT137230 | Canada | British Columbia |  |  |
| *M. californica pluviale* | HLC-23292 | KT147750 | Canada | British Columbia |  |  |
| *M. californica pluviale* | PFC-2006-0786 | n/a | Canada | British Columbia |  |  |
| *M. californica pluviale* | MNBTT-911 | KT135962 | Canada | New Brunswick |  |  |
| *M. californica pluviale* | CCDB-19575-C11 | n/a | United States | Arizona |  |  |
| *M. constricta* | 06-BLLOC-2772 | n/a | United States | California |  |  |
| *M. constricta* | 06-BLLOC-2996 | n/a | United States | California |  |  |
| *M. constricta* | 06-BLLOC-2997 | n/a | United States | California |  |  |
| *M. constricta* | 06-BLLOC-2998 | n/a | United States | California |  |  |
| *M. constricta* | 06-BLLOC-2999 | n/a | United States | California |  |  |
| *M. constricta* | 06-BLLOC-3000 | n/a | United States | California |  |  |
| *M. constricta* | 06-BLLOC-3001 | n/a | United States | California |  |  |
| *M. constricta* | 06-BLLOC-3002 | n/a | United States | California |  |  |
| *M. constricta* | 06-BLLOC-3003 | n/a | United States | California |  |  |
| *M. constricta* | 06-BLLOC-3004 | n/a | United States | California |  |  |
| *M. constricta* | 06-BLLOC-3005 | n/a | United States | California |  |  |
| *M. constricta* | 06-BLLOC-3243 | n/a | United States | California |  |  |
| *M. constricta* | 06-BLLOC-3244 | n/a | United States | California |  |  |
| *M. constricta* | 06-BLLOC-3245 | n/a | United States | California |  |  |
| *M. constricta* | 06-BLLOC-3246 | n/a | United States | California |  |  |
| *M. constricta* | BIOUG06805-B06 | n/a | United States | California |  |  |
| *M. constricta* | BIOUG06805-B07 | n/a | United States | California |  |  |
| *M. constricta* | BIOUG06805-B08 | n/a | United States | California |  |  |
| *M. constricta* | BIOUG06805-B09 | n/a | United States | California |  |  |
| *M. constricta* | BIOUG06805-B10 | n/a | United States | California |  |  |
| *M. constricta* | CNCLEP00063091 | GU678961 | United States | California |  |  |
| *M. constricta* | CNCLEP00063092 | GU678970 | United States | California |  |  |
| *M. disstria* | BIOUG03567-G01 | KM550592 | Canada | Alberta | 49.11 | -113.82 |
| *M. disstria* | BIOUG03610-F10 | KM547975 | Canada | Alberta | 49.08 | -113.88 |
| *M. disstria* | 08BBLEP-02575 | KM539652 | Canada | Alberta | 49.08 | -113.88 |
| *M. disstria* | 08BBLEP-02576 | KM553917 | Canada | Alberta | 49.08 | -113.88 |
| *M. disstria* | 08BBLEP-02578 | KM544964 | Canada | Alberta | 49.08 | -113.88 |
| *M. disstria* | 08BBLEP-02588 | KM553230 | Canada | Alberta | 49.08 | -113.88 |
| *M. disstria* | 08BBLEP-03307 | KM548321 | Canada | Alberta | 49.05 | -113.91 |
| *M. disstria* | 08BBLEP-03335 | KM544855 | Canada | Alberta | 49.05 | -113.91 |
| *M. disstria* | 08BBLEP-04315 | KM542660 | Canada | Alberta | 49.11 | -113.84 |
| *M. disstria* | 08BBLEP-04366 | KM544414 | Canada | Alberta | 49.11 | -113.84 |
| *M. disstria* | 08BBLEP-04893 | KM544027 | Canada | Alberta | 49.08 | -113.88 |
| *M. disstria* | 08BBLEP-04924 | KM551932 | Canada | Alberta | 49.08 | -113.88 |
| *M. disstria* | 08BBLEP-04925 | KM544829 | Canada | Alberta | 49.08 | -113.88 |
| *M. disstria* | 08BBLEP-04926 | KM545548 | Canada | Alberta | 49.08 | -113.88 |
| *M. disstria* | 08BBLEP-04931 | KM544076 | Canada | Alberta | 49.08 | -113.88 |
| *M. disstria* | 08BBLEP-04934 | KM552722 | Canada | Alberta | 49.08 | -113.88 |
| *M. disstria* | 08BBLEP-04935 | KM550898 | Canada | Alberta | 49.08 | -113.88 |
| *M. disstria* | 08BBLEP-04939 | KM551626 | Canada | Alberta | 49.08 | -113.88 |
| *M. disstria* | 08BBLEP-02277 | KM553907 | Canada | Alberta | 49.08 | -113.88 |
| *M. disstria* | 08BBLEP-02316 | KM542855 | Canada | Alberta | 49.08 | -113.88 |
| *M. disstria* | 10BBCLP-0424 | JF841460 | Canada | British Columbia | 50.99 | -118.16 |
| *M. disstria* | 10BBCLP-0429 | JF841465 | Canada | British Columbia | 50.63 | -116.06 |
| *M. disstria* | 10BBCLP-0430 | JF841466 | Canada | British Columbia | 50.99 | -118.16 |
| *M. disstria* | 10BBCLP-0434 | JF841470 | Canada | British Columbia | 50.99 | -118.16 |
| *M. disstria* | HLC-20368 | KT133104 | Canada | British Columbia | 51.41 | -117.48 |
| *M. disstria* | HLC-21131 | KT128472 | Canada | British Columbia | 51.02 | -118.21 |
| *M. disstria* | HLC-21135 | KT138178 | Canada | British Columbia | 51.02 | -118.21 |
| *M. disstria* | HLC-21137 | KT138487 | Canada | British Columbia | 51.02 | -118.21 |
| *M. disstria* | HLC-21138 | KT147660 | Canada | British Columbia | 51.02 | -118.21 |
| *M. disstria* | HLC-21875 | KT138260 | Canada | British Columbia | 51.09 | -117.92 |
| *M. disstria* | CGWC-0495 | KT144816 | Canada | British Columbia | 51.95 | -122.40 |
| *M. disstria* | CGWC-0501 | KT133205 | Canada | British Columbia | 51.92 | -122.29 |
| *M. disstria* | CGWC-0503 | KT141078 | Canada | British Columbia | 52.58 | -122.20 |
| *M. disstria* | CGWC-0504 | KT145295 | Canada | British Columbia | 52.58 | -122.20 |
| *M. disstria* | CGWC-0505 | KT125112 | Canada | British Columbia | 52.58 | -122.20 |
| *M. disstria* | CGWC-0506 | KT136401 | Canada | British Columbia | 52.58 | -122.20 |
| *M. disstria* | BIOUG03567-F11 | KM547067 | Canada | Manitoba | 50.68 | -99.90 |
| *M. disstria* | moth58.02SA | GU438466 | Canada | New Brunswick | 45.08 | -67.07 |
| *M. disstria* | moth87.02SA | GU438467 | Canada | New Brunswick | 45.08 | -67.07 |
| *M. disstria* | 04HBL00794 | GU438468 | Canada | New Brunswick | 45.08 | -67.07 |
| *M. disstria* | Moth 412.03SA | GU438464 | Canada | New Brunswick | 45.08 | -67.07 |
| *M. disstria* | Moth 459.03SA | GU438465 | Canada | New Brunswick | 45.08 | -67.07 |
| *M. disstria* | MNBTT-3236 | KT140062 | Canada | New Brunswick | 45.92 | -66.63 |
| *M. disstria* | MNBTT-3237 | KT142418 | Canada | New Brunswick | 45.92 | -66.63 |
| *M. disstria* | MNBTT-3238 | KT147875 | Canada | New Brunswick | 45.92 | -66.63 |
| *M. disstria* | MNBTT-3239 | KT144563 | Canada | New Brunswick | 45.92 | -66.63 |
| *M. disstria* | MNBTT-3240 | KT127281 | Canada | New Brunswick | 45.92 | -66.63 |
| *M. disstria* | MNBTT-908 | KT141800 | Canada | New Brunswick | 46.00 | -66.18 |
| *M. disstria* | MNBTT-909 | KT127657 | Canada | New Brunswick | 46.00 | -66.18 |
| *M. disstria* | 09BBELE-0267 | GU690177 | Canada | Nova Scotia | 46.81 | -60.77 |
| *M. disstria* | 09BBELE-0302 | GU690329 | Canada | Nova Scotia | 46.81 | -60.77 |
| *M. disstria* | 09BBELE-0598 | HM414890 | Canada | Nova Scotia | 46.83 | -60.61 |
| *M. disstria* | 09BBELE-0284 | GU690161 | Canada | Nova Scotia | 46.81 | -60.77 |
| *M. disstria* | BIOUG09673-F10 | KR450034 | Canada | Ontario | 44.85 | -79.87 |
| *M. disstria* | BIOUG09852-C06 | KR445802 | Canada | Ontario | 44.85 | -79.87 |
| *M. disstria* | BIOUG10646-F02 | KR449825 | Canada | Ontario | 44.85 | -79.87 |
| *M. disstria* | BIOUG10646-F03 | KR449567 | Canada | Ontario | 44.85 | -79.87 |
| *M. disstria* | BIOUG10646-F04 | KR451437 | Canada | Ontario | 44.85 | -79.87 |
| *M. disstria* | BIOUG10646-F05 | KR449337 | Canada | Ontario | 44.85 | -79.87 |
| *M. disstria* | BIOUG11791-F05 | KR453939 | Canada | Ontario | 44.85 | -79.87 |
| *M. disstria* | BIOUG11791-F06 | KR449198 | Canada | Ontario | 44.85 | -79.87 |
| *M. disstria* | moth994.01 | GU094644 | Canada | Ontario | 43.54 | -80.13 |
| *M. disstria* | BIOUG22324-G02 | KT623013 | Canada | Ontario | 43.37 | -80.36 |
| *M. disstria* | BIOUG22569-B02 | KT622571 | Canada | Ontario | 43.37 | -80.36 |
| *M. disstria* | BIOUG22569-B03 | KT622526 | Canada | Ontario | 43.37 | -80.36 |
| *M. disstria* | BIOUG22569-B04 | KT619741 | Canada | Ontario | 43.37 | -80.36 |
| *M. disstria* | BIOUG22569-B05 | KT621843 | Canada | Ontario | 43.37 | -80.36 |
| *M. disstria* | BIOUG21896-A08 | KT622901 | Canada | Ontario | 43.37 | -80.35 |
| *M. disstria* | 04HBL005114 | GU094067 | Canada | Ontario | 43.54 | -80.13 |
| *M. disstria* | 04HBL005145 | GU094069 | Canada | Ontario | 43.54 | -80.13 |
| *M. disstria* | 04HBL005146 | GU094068 | Canada | Ontario | 43.54 | -80.13 |
| *M. disstria* | 04HBL005206 | GU094070 | Canada | Ontario | 43.54 | -80.13 |
| *M. disstria* | 2005-ONT-554 | GU438997 | Canada | Ontario | 44.53 | -77.00 |
| *M. disstria* | 2005-ONT-560 | GU438998 | Canada | Ontario | 44.53 | -77.00 |
| *M. disstria* | 2005-ONT-561 | GU438999 | Canada | Ontario | 44.53 | -77.00 |
| *M. disstria* | 2005-ONT-569 | GU439001 | Canada | Ontario | 44.53 | -77.00 |
| *M. disstria* | 2005-ONT-570 | GU439003 | Canada | Ontario | 44.53 | -77.00 |
| *M. disstria* | 2005-ONT-571 | GU439002 | Canada | Ontario | 44.53 | -77.00 |
| *M. disstria* | 2005-ONT-572 | GU439000 | Canada | Ontario | 44.53 | -77.00 |
| *M. disstria* | 2005-ONT-578 | GU439005 | Canada | Ontario | 44.53 | -77.00 |
| *M. disstria* | 2005-ONT-579 | GU439004 | Canada | Ontario | 44.53 | -77.00 |
| *M. disstria* | Moth4379.03 | GU092468 | Canada | Ontario | 43.54 | -80.13 |
| *M. disstria* | Moth4416.03 | GU092469 | Canada | Ontario | 43.54 | -80.13 |
| *M. disstria* | Moth4432.03 | GU092470 | Canada | Ontario | 43.54 | -80.13 |
| *M. disstria* | Moth4516.03 | GU092471 | Canada | Ontario | 43.54 | -80.13 |
| *M. disstria* | Moth4603.03 | GU092472 | Canada | Ontario | 43.54 | -80.13 |
| *M. disstria* | 2006-ONT-0835 | KT141948 | Canada | Ontario | 44.53 | -77.00 |
| *M. disstria* | 2006-ONT-0893 | KT134186 | Canada | Ontario | 44.53 | -77.00 |
| *M. disstria* | 2006-ONT-0920 | KT147609 | Canada | Ontario | 44.53 | -77.00 |
| *M. disstria* | 2006-ONT-1441 | KT148152 | Canada | Ontario | 44.53 | -77.00 |
| *M. disstria* | 2006-ONT-1442 | KT126541 | Canada | Ontario | 44.53 | -77.00 |
| *M. disstria* | BIOUG06756-F07 | n/a | Canada | Ontario | 45.52 | -78.42 |
| *M. disstria* | BIOUG06756-D09 | n/a | Canada | Ontario | 45.46 | -78.80 |
| *M. disstria* | BL606 | n/a | Canada | Ontario | n/a | n/a |
| *M. disstria* | BL696 | n/a | Canada | Ontario | n/a | n/a |
| *M. disstria* | BL816 | n/a | Canada | Ontario | n/a | n/a |
| *M. disstria* | LEP041295 | n/a | Canada | Ontario | n/a | n/a |
| *M. disstria* | LEP041296 | n/a | Canada | Ontario | n/a | n/a |
| *M. disstria* | BIOUG23415-H08 | n/a | Canada | Ontario | 43.37 | -80.36 |
| *M. disstria* | BIOUG23203-C08 | n/a | Canada | Ontario | 43.37 | -80.35 |
| *M. disstria* | DH001568 | n/a | Canada | Quebec | 45.69 | -73.09 |
| *M. disstria* | DH005298 | n/a | Canada | Quebec | n/a | n/a |
| *M. disstria* | BIOUG03567-H08 | KM548159 | Canada | Saskatchewan | 53.85 | -106.08 |
| *M. disstria* | BIOUG03567-H09 | KM543700 | Canada | Saskatchewan | 53.85 | -106.08 |
| *M. disstria* | BIOUG03567-H10 | KM549781 | Canada | Saskatchewan | 53.85 | -106.08 |
| *M. disstria* | BIOUG03567-H11 | KM548219 | Canada | Saskatchewan | 53.85 | -106.08 |
| *M. disstria* | BIOUG04567-A02 | KM546539 | Canada | Saskatchewan | 53.85 | -106.08 |
| *M. disstria* | BIOUG04567-A06 | KM549713 | Canada | Saskatchewan | 53.85 | -106.08 |
| *M. disstria* | BIOUG04566-A07 | KM551575 | Canada | Saskatchewan | 53.85 | -106.08 |
| *M. disstria* | BIOUG04566-A08 | KM546418 | Canada | Saskatchewan | 53.85 | -106.08 |
| *M. disstria* | 10BBLEP-00512 | HQ985859 | United States | Arkansas | 35.08 | -92.55 |
| *M. disstria* | 10BBLEP-00513 | HQ985860 | United States | Arkansas | 35.37 | -93.34 |
| *M. disstria* | 10BBLEP-00514 | n/a | United States | Arkansas | 35.37 | -93.34 |
| *M. disstria* | 10BBLEP-00515 | HQ985861 | United States | Arkansas | 35.37 | -93.34 |
| *M. disstria* | CNCNoctuoidea13836 | n/a | United States | Florida | n/a | n/a |
| *M. disstria* | 06-JKA-0702 | n/a | United States | Georgia | n/a | n/a |
| *M. disstria* | 06-SUSA-0166 | n/a | United States | Kentucky | 37.01 | -88.54 |
| *M. disstria* | 06-SUSA-0173 | n/a | United States | Kentucky | 37.01 | -88.54 |
| *M. disstria* | 06-SUSA-0175 | n/a | United States | Kentucky | 37.01 | -88.54 |
| *M. disstria* | 06-NCCC-1094 | n/a | United States | North Carolina | 34.77 | -76.76 |
| *M. disstria* | 06-NCCC-1095 | n/a | United States | North Carolina | 34.77 | -76.76 |
| *M. disstria* | 06-NCCC-1096 | n/a | United States | North Carolina | 34.77 | -76.76 |
| *M. disstria* | MDOK-2057 | GU800045 | United States | Oklahoma | 36.74 | -95.95 |
| *M. disstria* | MDOK-1495 | n/a | United States | Oklahoma | 36.74 | -95.95 |
| *M. disstria* | MDOK-2368 | GU801093 | United States | Oklahoma | 36.74 | -95.95 |
| *M. disstria* | MDOK-2411 | GU801051 | United States | Oklahoma | 36.74 | -95.95 |
| *M. disstria* | MDOK-2415 | GU801047 | United States | Oklahoma | 36.74 | -95.95 |
| *M. disstria* | BIOUG02884-A02 | n/a | United States | Tennessee | 35.69 | -83.50 |
| *M. disstria* | BIOUG02884-A03 | n/a | United States | Tennessee | 35.69 | -83.50 |
| *M. disstria* | BIOUG02884-A04 | n/a | United States | Tennessee | 35.69 | -83.50 |
| *M. disstria* | BIOUG02884-A05 | n/a | United States | Tennessee | 35.69 | -83.50 |
| *M. disstria* | BIOUG02884-A07 | n/a | United States | Tennessee | 35.69 | -83.50 |
| *M. disstria* | BIOUG02884-A11 | n/a | United States | Tennessee | 35.69 | -83.50 |
| *M. disstria* | BIOUG03567-H02 | n/a | United States | Tennessee | 35.69 | -83.50 |
| *M. disstria* | BIOUG03567-H04 | n/a | United States | Tennessee | 35.69 | -83.50 |
| *M. disstria* | BIOUG03567-H05 | n/a | United States | Tennessee | 35.69 | -83.50 |
| *M. disstria* | BIOUG03567-H07 | n/a | United States | Tennessee | 35.69 | -83.50 |
| *M. disstria* | TAMUICEGR-0827 | HQ978007 | United States | Texas | 30.59 | -96.25 |
| *M. disstria* | TAMUICEGR-0828 | HQ978008 | United States | Texas | 30.59 | -96.25 |
| *M.incurva* | CMAZ-0343 | JF846748 | United States | Arizona |  |  |
| *M.incurva* | CMAZ-0344 | JF846749 | United States | Arizona |  |  |
| *M.incurva* | CMAZ-0345 | JF846750 | United States | Arizona |  |  |
| *M.incurva* | CCDB-22945-F01 | n/a | United States | Arizona |  |  |
| *M.incurva* | CCDB-22945-E12 | n/a | United States | Arizona |  |  |
