# Supplementary material for: Phylogeographic structure in three North American tent caterpillar species (Lepidoptera: Lasiocampidae): Malacosoma americana, M. californica, and M. disstria
Source: PeerJ. 2018 Mar 19;6:e4479. doi: 10.7717/peerj.4479 (PMC5863710; doi:10.7717/peerj.4479)
Supplement: Table S2 — Sample size (n) and distribution of haplotypes (h), haplotype diversity (Hd), and nucleotide diversity (π) among the (A) M. americana, (B) M. californica, and (C) M. disstria sampling locations. Shared haplotypes have been allocated a letter. The allocation of samples into Bayesian clusters (BAPS) is also given. [file peerj-06-4479-s002.docx]

| (a) | NB | QC | ON | MN | KY | IL | NC | MD | TN | TX | OK | AR | Total |
| --- | --- | --- | --- | --- | --- | --- | --- | --- | --- | --- | --- | --- | --- |
| A |  |  | 16 |  |  |  | 1 | 1 |  |  |  |  | **18** |
| B |  |  | 10 |  |  |  |  |  |  |  |  |  | **10** |
| C | 8 |  |  |  |  |  |  |  |  |  |  |  | **8** |
| D | 1 |  | 4 |  |  |  |  |  |  |  |  |  | **5** |
| E |  |  |  | 1 |  |  |  |  |  |  | 4 |  | **5** |
| F |  |  |  |  |  |  |  |  |  |  | 3 |  | **3** |
| G |  |  |  |  |  |  |  |  |  | 2 |  |  | **2** |
| H |  |  |  |  |  | 1 |  |  |  |  | 1 |  | **2** |
| I |  |  |  |  | 1 |  |  |  | 1 |  |  |  | **2** |
| # unique | 3 | 1 | 9 | 2 |  |  | 1 |  | 3 | 2 | 1 | 2 | **24** |
| n | **12** | **1** | **39** | **3** | **1** | **1** | **2** | **1** | **4** | **4** | **9** | **2** | **79** |
| h | **5** | **1** | **12** | **3** | **1** | **1** | **2** | **1** | **4** | **3** | **4** | **2** | **33** |
| Hd | 0.576 | n/a | 0.769 | 1.000 | n/a | n/a | 1.000 | n/a | 1.000 | 0.833 | 0.750 | 1.000 | **0.918** |
| π | 0.0013 | n/a | 0.0027 | 0.0061 | n/a | n/a | 0.0046 | n/a | 0.0061 | 0.0058 | 0.0041 | 0.0046 | **0.0049** |
| BAPS 1 | 12 | 1 | 28 |  |  | 1 | 1 |  | 1 |  | 1 |  | **45** |
| BAPS 2 |  |  | 10 | 1 | 1 |  | 1 | 1 | 3 | 4 | 5 | 2 | **28** |
| BAPS 3 |  |  | 1 | 2 |  |  |  |  |  |  | 3 |  | **6** |

| (b) | **cBC** | **eBC** | **swBC** | **VI** | **WA** | **CA** | **sCA** | **AZ** | **TX** | **Total** |
| --- | --- | --- | --- | --- | --- | --- | --- | --- | --- | --- |
| **A** |  | 44 |  |  |  |  |  |  |  | **44** |
| **B** | 4 | 18 |  |  |  |  |  |  |  | **22** |
| **C** |  | 18 |  |  |  |  |  |  |  | **18** |
| **D** |  | 8 | 2 | 5 |  |  |  |  |  | **15** |
| **E** |  | 14 |  |  |  |  |  |  |  | **14** |
| **F** |  |  |  | 7 | 1 |  |  |  |  | **8** |
| **G** |  | 5 |  |  |  |  |  |  |  | **5** |
| **H** |  |  | 1 | 4 |  |  |  |  |  | **5** |
| **I** |  | 3 |  |  |  |  |  |  |  | **3** |
| **J** |  |  |  | 3 |  |  |  |  |  | **3** |
| **K** |  |  |  |  |  |  | 3 |  |  | **3** |
| **L** |  |  |  |  |  |  |  | 3 |  | **3** |
| **M** |  |  |  |  |  |  |  | 2 |  | **2** |
| **N** |  | 2 |  |  |  |  |  |  |  | **2** |
| **O** |  | 2 |  |  |  |  |  |  |  | **2** |
| **P** |  | 2 |  |  |  |  |  |  |  | **2** |
| **Q** |  | 2 |  |  |  |  |  |  |  | **2** |
| **R** |  | 2 |  |  |  |  |  |  |  | **2** |
| **S** |  | 2 |  |  |  |  |  |  |  | **2** |
| **T** | 2 |  |  |  |  |  |  |  |  | **2** |
| **U** |  |  | 2 |  |  |  |  |  |  | **2** |
| **V** |  |  |  | 2 |  |  |  |  |  | **2** |
| **W** |  |  |  |  | 2 |  |  |  |  | **2** |
| **X** |  |  |  |  |  | 1 |  |  | 1 | **2** |
| **# unique** | 3 | 22 |  | 4 |  | 5 | 1 | 5 |  | **40** |
| **n** | **9** | **144** | **5** | **25** | **3** | **6** | **4** | **10** | **1** | **207** |
| **h** | **5** | **35** | **3** | **9** | **2** | **6** | **2** | **7** | **1** | **64** |
| **Hd** | 0.806 | 0.865 | 0.800 | 0.863 | 0.667 | 1.000 | 0.500 | 0.911 | n/a | **0.925** |
| **π** | 0.0036 | 0.0077 | 0.0033 | 0.0024 | 0.0020 | 0.0071 | 0.0015 | 0.0093 | n/a | **0.0097** |
| **BAPS 1** |  | 53 |  |  |  |  |  |  |  | **53** |
| **BAPS 2** |  | 27 |  |  |  | 4 |  |  |  | **31** |
| **BAPS 3** | 1 | 23 |  | 2 |  |  |  |  |  | **26** |
| **BAPS 4** | 8 | 41 | 5 | 23 | 3 | 2 |  |  | 1 | **83** |
| **BAPS 5** |  |  |  |  |  |  |  | 10 |  | **10** |
| **BAPS 6** |  |  |  |  |  |  | 4 |  |  | **4** |

| (c) | **CBC** | **eBC** | **AB** | **SK** | **MB** | **cON** | **sON** | **eON** | **QC** | **NB** | **NS** | **TX** | **OK** | **AR** | **KY** | **TN** | **NC** | **GA** | **FL** | **Total** |
| --- | --- | --- | --- | --- | --- | --- | --- | --- | --- | --- | --- | --- | --- | --- | --- | --- | --- | --- | --- | --- |
| **A** |  |  |  |  |  | 5 | 5 | 9 | 1 | 3 | 3 |  |  |  |  |  |  |  |  | **26** |
| **B** |  |  | 8 | 8 | 1 |  |  |  |  |  |  |  |  |  |  |  |  |  |  | **17** |
| **C** | 4 | 9 | 1 |  |  |  |  |  |  |  |  |  |  |  |  |  |  |  |  | **14** |
| **D** |  |  |  |  |  |  |  | 1 |  | 4 |  |  | 2 | 1 |  | 2 | 1 | 1 |  | **12** |
| **E** |  |  |  |  |  | 3 | 2 | 5 |  |  |  |  |  |  |  |  |  |  |  | **10** |
| **F** |  |  |  |  |  |  |  |  |  |  |  |  |  |  |  | 6 |  |  |  | **6** |
| **G** |  |  | 4 |  |  |  | 1 |  |  |  |  |  |  |  |  |  |  |  |  | **5** |
| **H** |  |  |  |  |  |  |  | 2 |  |  |  |  | 1 |  | 2 |  |  |  |  | **5** |
| **I** |  |  |  |  |  |  | 4 |  |  |  |  |  |  |  |  |  |  |  |  | **4** |
| **J** |  |  | 3 |  |  |  |  |  |  |  |  |  |  |  |  |  |  |  |  | **3** |
| **K** |  |  | 2 |  |  |  |  |  |  |  |  |  |  |  |  |  |  |  |  | **2** |
| **L** |  |  |  |  |  |  | 2 |  |  |  |  |  |  |  |  |  |  |  |  | **2** |
| **M** |  |  |  |  |  |  |  | 2 |  |  |  |  |  |  |  |  |  |  |  | **2** |
| **N** |  |  |  |  |  |  | 1 |  | 1 |  |  |  |  |  |  |  |  |  |  | **2** |
| **O** |  |  |  |  |  |  |  |  |  |  |  |  |  | 2 |  |  |  |  |  | **2** |
| **# unique** | 2 | 1 | 2 |  |  |  | 4 | 1 |  | 5 | 1 | 2 | 2 | 1 | 1 | 2 | 2 |  | 1 | **27** |
| **n** | **6** | **10** | **20** | **8** | **1** | **8** | **19** | **20** | **2** | **12** | **4** | **2** | **5** | **4** | **3** | **10** | **3** | **1** | **1** | **139** |
| **h** | **3** | **2** | **7** | **1** | **1** | **2** | **10** | **6** | **2** | **7** | **2** | **2** | **4** | **3** | **2** | **4** | **3** | **1** | **1** | **42** |
| **Hd** | 0.600 | 0.200 | 0.800 | 0.000 | n/a | 0.536 | 0.895 | 0.747 | 1.000 | 0.864 | 0.500 | 1.000 | 0.900 | 0.833 | 0.667 | 0.644 | 1.000 | n/a | n/a | **0.926** |
| **π** | 0.0010 | 0.0003 | 0.0035 | 0.000 | n/a | 0.0065 | 0.0053 | 0.0065 | 0.0015 | 0.0033 | 0.0023 | 0.0137 | 0.0030 | 0.0046 | 0.0010 | 0.0013 | 0.0030 | n/a | n/a | **0.0063** |
| **BAPS 1** |  |  | 4 |  |  |  | 6 | 3 |  | 4 | 1 | 1 | 5 | 4 | 3 | 10 | 3 | 1 | 1 | **46** |
| **BAPS 2** |  |  |  |  |  | 5 | 10 | 10 | 2 | 4 | 3 |  |  |  |  |  |  |  |  | **34** |
| **BAPS 3** | 6 | 10 | 1 |  |  | 3 | 2 | 7 |  |  |  | 1 |  |  |  |  |  |  |  | **30** |
| **BAPS 4** |  |  | 15 | 8 | 1 |  | 1 |  |  | 4 |  |  |  |  |  |  |  |  |  | **29** |
