# Supplementary figures and images for: Phylogeographic structure in three North American tent caterpillar species (Lepidoptera: Lasiocampidae): Malacosoma americana, M. californica, and M. disstria
Source: PeerJ. 2018 Mar 19;6:e4479. doi: 10.7717/peerj.4479 (PMC5863710; doi:10.7717/peerj.4479)

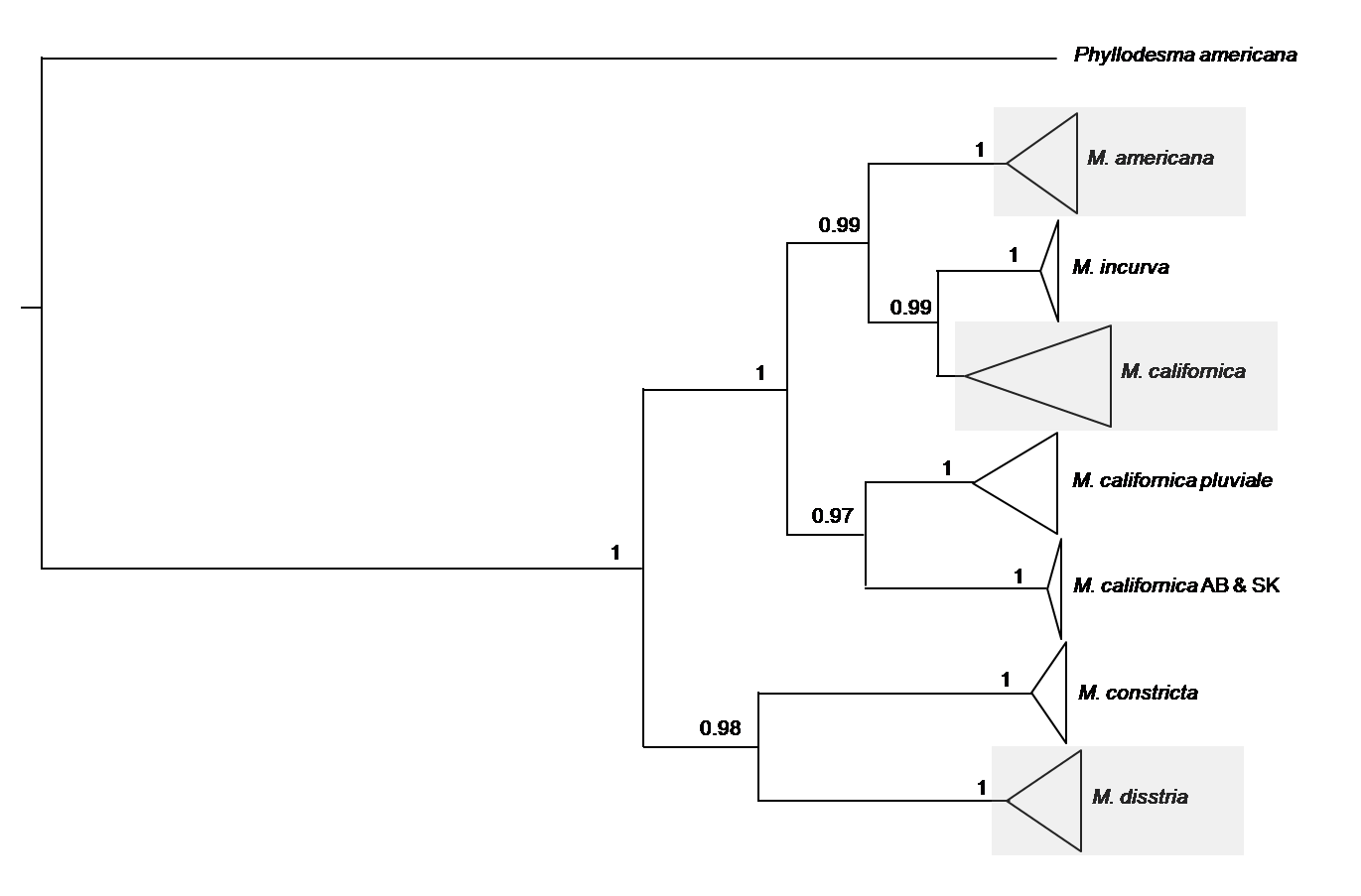

Supplement: Figure S1 — Bayesian analysis based on 474 COI sequences from five North American species of Malacosoma. The triangles represent multiple specimens from the same species, with the length of the triangle representative of the sequence variation. Posterior values ¿0.8 are given. The shaded boxes represent the samples used in this study; further detail is given in Figs. S3–S5. [file peerj-06-4479-s003.png]

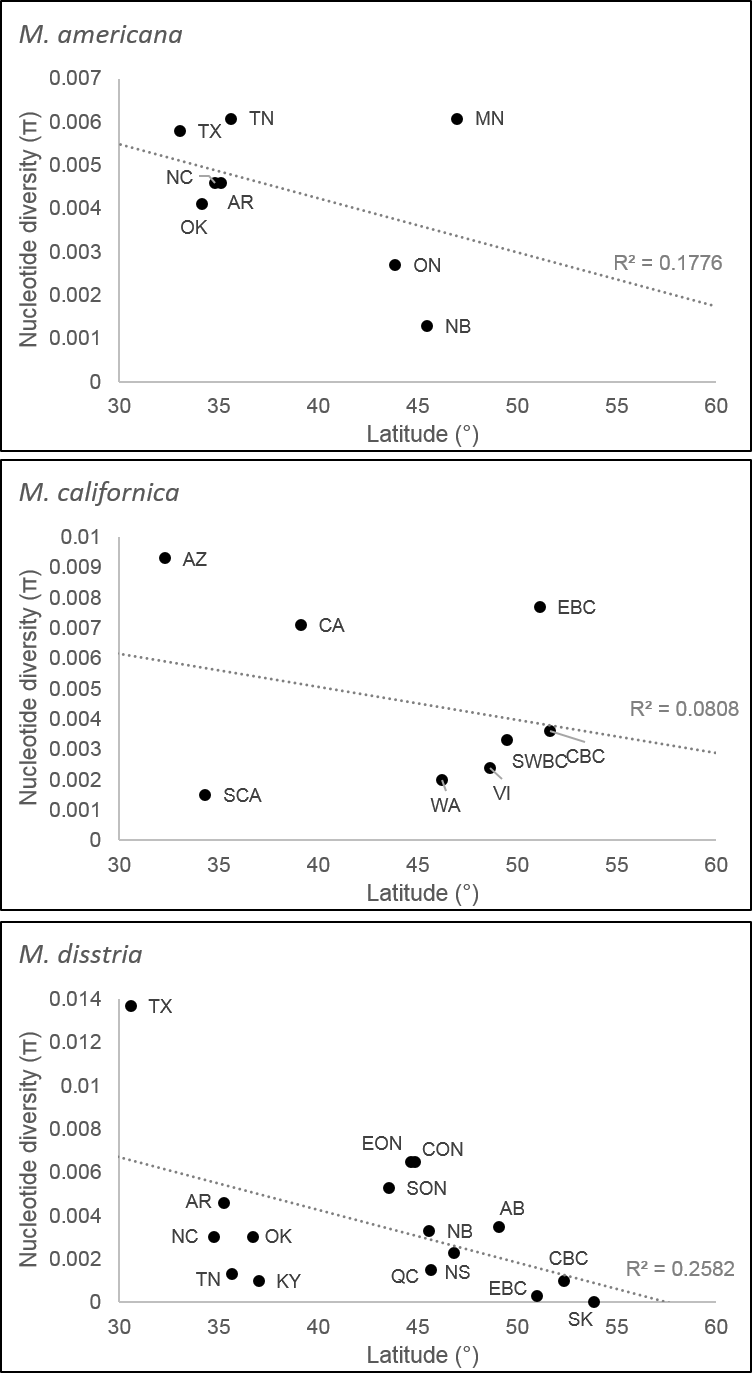

Supplement: Figure S2 — Comparison of diversity relative to latitude for M. americana, M. californica, and M. disstria. The correlation coefficients were −0.421, −0.284, and −0.508, respectively. All populations in this study above 40°N are in previously glaciated regions. [file peerj-06-4479-s004.png]

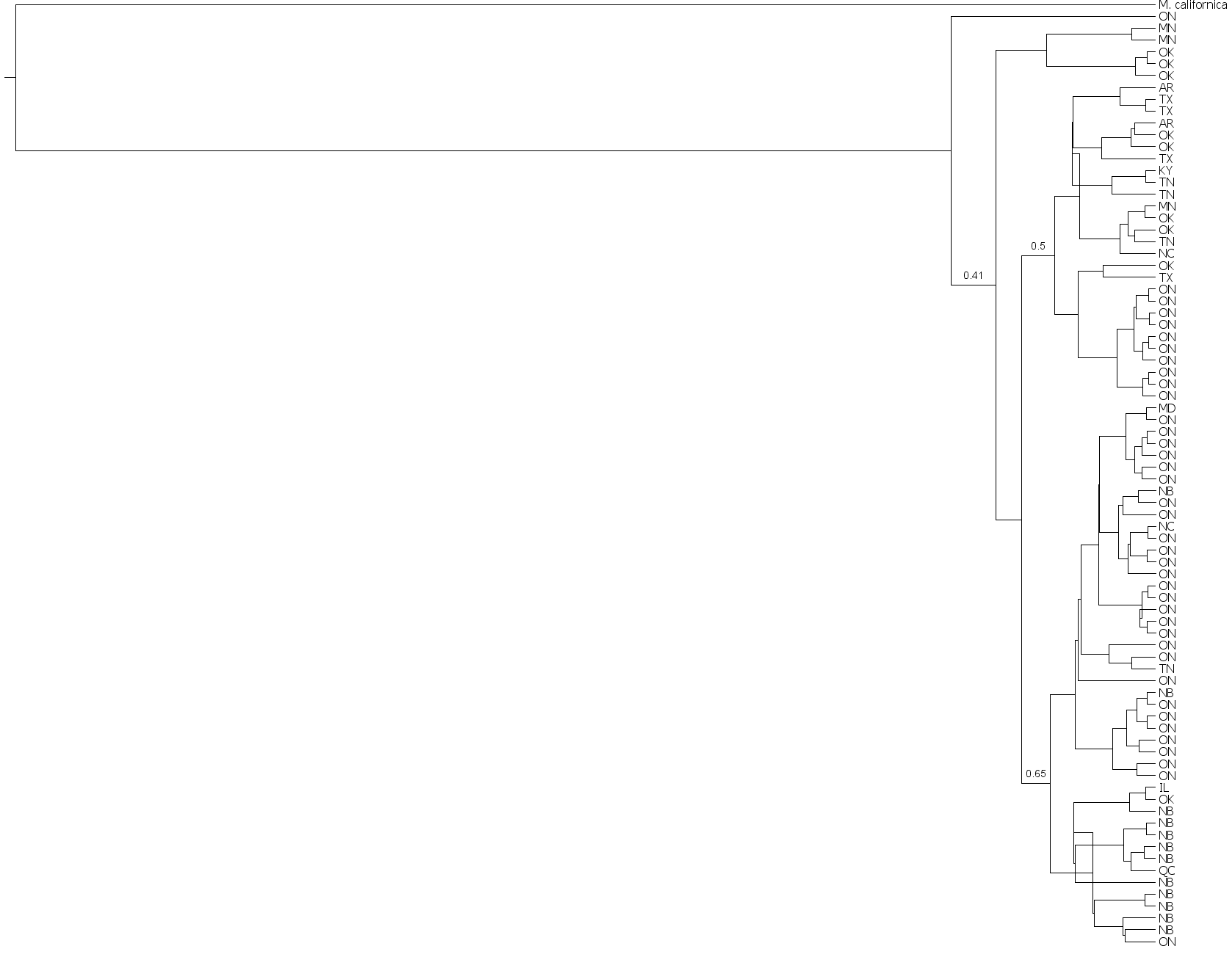

Supplement: Figure S3 — Bayesian analysis based on 79 M. americana sequences and rooted with the congeneric M. californica. Posterior values are given for major clades. Tree files are available upon request. [file peerj-06-4479-s005.png]

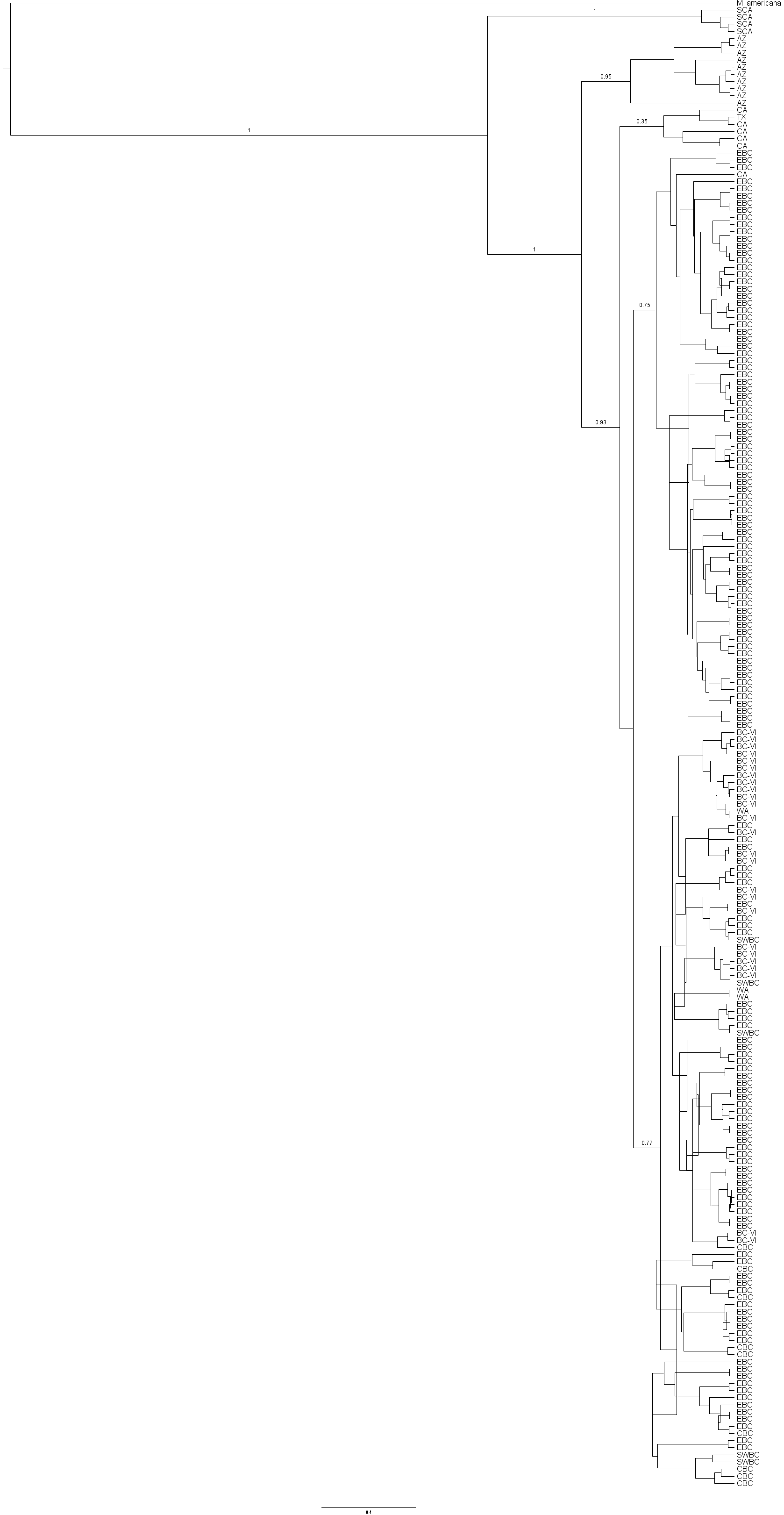

Supplement: Figure S4 — Bayesian analysis based on 207 M. californica sequences and rooted with the congeneric M. americana. Posterior values are given for major clades. Tree files are available upon request. [file peerj-06-4479-s006.png]

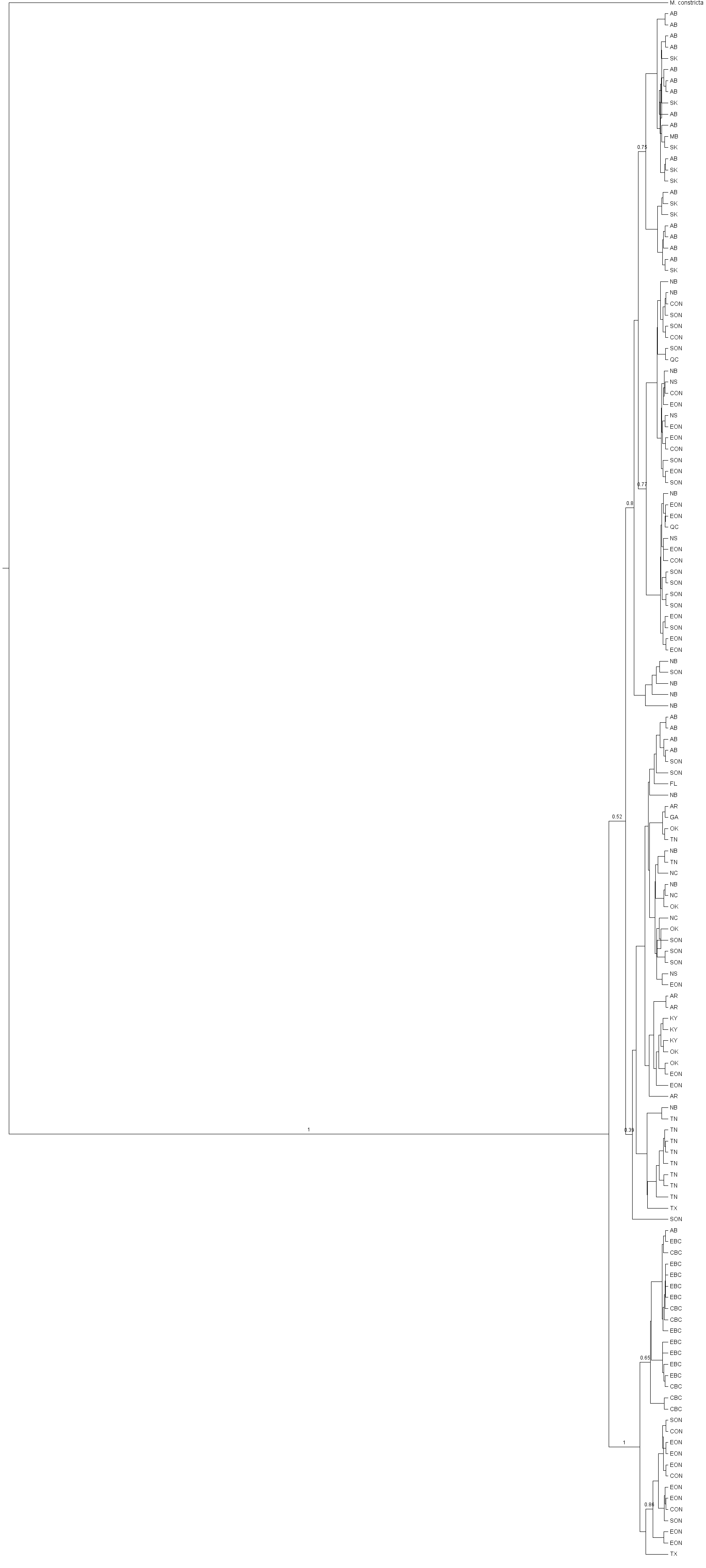

Supplement: Figure S5 — Bayesian analysis based on139 M. disstria sequences and rooted with the congeneric M. constricta. Posterior values are given for major clades. Tree files are available upon request. [file peerj-06-4479-s007.png]
